# Epidemiology of hepatitis C virus infection among people receiving opioid substitution therapy (ECHO): study protocol

**DOI:** 10.1186/s12879-015-1307-z

**Published:** 2015-12-10

**Authors:** Lisa Strada, Bernd Schulte, Christiane Sybille Schmidt, Uwe Verthein, Peter Cremer-Schaeffer, Sabine Krückeberg, Jens Reimer

**Affiliations:** Centre for Interdisciplinary Addiction Research, University Medical Centre Hamburg-Eppendorf, Martinistr. 52, 20246 Hamburg, Germany; Federal Institute for Drugs and Medical Devices (BfArM), Kurt-Georg-Kiesinger-Allee 3, 53175 Bonn, Germany

**Keywords:** Hepatitis C virus (HCV), Opioid substitution therapy (OST), Prevalence, Incidence, Treatment uptake, Seroconversion

## Abstract

**Background:**

Hepatitis C virus infection is highly prevalent among people who inject drugs. Opioid substitution therapy, the standard treatment for opioid dependence, provides an excellent opportunity for the treatment of hepatitis C virus infection due to the close and regular contact between patients and clinicians. However, there is little research on the impact of opioid substitution therapy on the prevalence of the hepatitis C virus at a national level. This paper describes the protocol for the Epidemiology of Hepatitis C Virus Infection among People Receiving Opioid Substitution Therapy (ECHO) study. The aim of this study is to estimate the national prevalence and incidence of hepatitis C virus infection among people receiving opioid substitution therapy in Germany and to describe factors associated with hepatitis C treatment uptake and seroconversion.

**Methods/Design:**

An observational, longitudinal, multicentre study is being conducted between 2014 and 2016 in a representative sample of approximately 2500 people receiving opioid substitution therapy from about 100 clinicians providing opioid substitution therapy in Germany. Data will be collected during routine patient care and by means of patient and clinician questionnaires at baseline and 12-month follow-up. Stratified sampling will be performed to obtain a representative sample of clinicians providing opioid substitution therapy. The strata will be constructed based on the distribution of the total sample of clinicians providing opioid substitution therapy in Germany according to German Federal State and the number of patients per clinician.

**Discussion:**

Opioid substitution therapy may be an important strategy to prevent the spread of hepatitis C virus in opioid dependent populations, but its effectiveness may be diminished by our limited understanding of factors associated with treatment uptake and seroconversion. The present study will provide important information for developing strategies to address hepatitis C virus-related disease burden in people receiving opioid substitution therapy.

**Trial registration:**

ClinicalTrials.gov: NCT02395198

## Background

Injecting drug use is the primary mode of transmission for hepatitis C virus (HCV) infection in developed countries [1–3] and accounts for the vast majority of new infections [4, 5]. With up to 90 % of people who inject drugs (PWID) being HCV-antibody-positive, HCV is highly prevalent in this target group. Worldwide, 6–15 million drug users are infected with the virus [6]. Opioid substitution therapy (OST), the standard treatment for opioid dependence, provides an excellent opportunity for managing HCV infection due to the close and regular contact between patients and clinicians. However, there is little research on the impact of OST on HCV prevalence and incidence at a national level.

HCV infection is characterized by an asymptomatic onset and progression, which delays early diagnosis and therefore treatment. Treating the infection in its early stages is vital to avoiding long-term complications. Approximately 15 % of chronic HCV-infected PWID develop liver cirrhosis within 20 years [7] and the burden of HCV-related morbidity and mortality among PWID is significant and increasing [8–10]. Infection duration is a risk factor for accelerated liver disease progression [1] and the stage of liver disease is, in turn, a predictor for the success of antiviral treatment [11, 12]. Early diagnosis of HCV infection is therefore of utmost importance.

Accordingly, the current guidelines of the Association of the Scientific Medical Societies in Germany (AWMF) for the prevention, diagnosis and treatment of HCV infections recommends testing former and active drug users every 12 months [13]. OST provides optimal conditions for regularly testing and monitoring HCV infections by virtue of the therapeutic relationship and routine contact between patients and clinicians. Nevertheless, recent data from a convenience sample of nearly 600 OST clinicians in Germany demonstrated limited implementation of the AMWF guidelines, particularly in small OST centres, with only two-thirds of the centres regularly testing patients’ HCV status [14].

In 2011–2012, HCV antibody levels among national samples of PWID in Europe varied from 19 to 84 %, with 7 of 11 countries reporting prevalence rates higher than 50 % [15]. Although no national data is available for Germany, subnational coverage in 2011 suggests a high prevalence of HCV, with 56 % of PWID in Berlin having ever been infected with HCV and 71.6 % in the city of Essen [16, 17]. Cohort studies provide some evidence for the effectiveness of OST in reducing HCV infections [18, 19]. Also model projections show that OST can reduce HCV prevalence if its coverage is increased sufficiently, however these reductions can be modest and require long-term coverage [20–22]. Real-life data on the effect of OST on HCV prevalence at a national level is lacking.

Despite growing evidence that PWID can successfully be treated for HCV [23], treatment uptake varies considerably between countries, from 0 to 64 % [24–26]. There are various barriers to HCV treatment initiation, including fears of low efficacy and adverse side effects among drug users, as well as concerns of low adherence and perceived risk of reinfection among clinicians [14, 27–29]. Yet our understanding of factors influencing HCV treatment uptake in OST is limited, as many of these studies have been conducted in populations of drug users rather than OST patients, using convenience samples and not representative national samples.

Moreover, the findings are based on the old HCV treatment regimen with interferon as opposed to newer treatments with direct-acting antivirals (DAA) without interferon. Concerns have been raised that drug users may become careless in safe injection practices if they are aware of the high success rate and few side effects of the new HCV therapy [30], thereby leading to higher reinfection rates. With the impending interferon-free era of HCV therapy, it is important to re-examine factors influencing treatment uptake and seroconversion to inform public health strategies.

The aim of the Epidemiology of Hepatitis C Virus Infection among People Receiving Opioid Substitution Therapy (ECHO) study is to estimate the national prevalence and incidence of HCV infection among OST patients in Germany and to examine factors associated with HCV treatment uptake and seroconversion.

## Methods/Design

### Study design

ECHO is a non-interventional, observational, longitudinal study being conducted between 2014 and 2016 in a large representative sample of approximately 2500 OST patients from about 100 OST clinicians across Germany. Data is collected during routine patient care and from questionnaires administered to patients and clinicians.

At baseline, we examine OST patients’ HCV status to assess HCV prevalence. Also the rate of HCV treatment uptake among OST patients and factors associated with treatment uptake are examined. Patients that are HCV negative at baseline or started HCV treatment up to 12 months prior to baseline are followed up after 12 months to assess the HCV incidence rate, factors influencing seroconversion, and (if applicable) the course of HCV treatment (e.g., treatment outcome, side effects). At both baseline and follow-up, the HCV status will be recorded from the participant’s chart. Clinicians are reminded of the HCV treatment guidelines in Germany, which recommend yearly HCV testing in this high-risk population, to encourage them to perform HCV antibody and/ or HCV RNA testing through the standard of care within the clinics. The study duration is either 1 day or 12 months depending on whether participants take part in the follow-up. Patient recruitment takes place over 12 months, data collection over 18 months, and the total length of the study is 27 months.

### Participants

#### Eligibility criteria

Study participants include patients and their clinicians. Clinicians are recruited first and must:

- Meet the minimum qualification requirements for addiction therapy in accordance with the Ordinance on the Prescription, Dispensing and Proof of the Disposition of Narcotic Drugs (BtMVV) as defined by the Chamber of Physicians
- Currently provide OST to 4 or more patients
- Have provided written informed consent

Patients recruited from the consenting clinicians must meet the following criteria:

- Diagnosed opioid dependence according to the International Statistical Classification of Diseases and Related Health Problems, 10^th^ revision (ICD-10; [31])
- Currently in OST
- Minimum age 18
- No severe mental impairment
- Sufficient German reading and writing skills
- Written informed consent

In Germany, clinicians that do not meet the requirements for addiction therapy in accordance with the BtMVV are allowed to provide OST to a maximum of three patients under the supervision of a clinician (consultant) meeting the minimum qualification requirements for addiction therapy. We exclude all clinicians with less than four OST patients to ensure that only clinicians with a qualification for addiction therapy are included in the study.

#### Sample size

The sample size calculation is based on the primary outcome variable of HCV incidence among OST patients. The literature reports an HCV incidence of 2–4 per 100 person-years in this target group [32, 33]. For a sample size of (at least) 32 patients with HCV seroconversion and an observation period of 1 year, 800 HCV antibody-negative patients are needed as an initial sample size. Based on previous literature [34], we assume an HCV antibody prevalence of about 68 % among OST patients in Germany. Therefore a sample size of 2500 patients is needed (32 % corresponds to *N* = 800).

### Recruitment

Obtaining a representative sample of OST patients is essential in determining the HCV prevalence and incidence among OST patients at a national level.

#### Clinician recruitment

In Germany, all clinicians who prescribe opioid substitution medication are registered in the national Substitution Register, a database maintained by the Federal Opium Agency of the Federal Institute for Drugs and Medical Devices (*Bundesinstitut für Arzneimittel und Medizinprodukte, BfArM*). In 2013, the Substitution Register counted 2691 clinicians prescribing opioid substitution medication. Information about clinicians in the Substitution Register is unavailable to the public. To ensure that all OST clinicians in Germany are considered in the present study, study invitations were sent to all substitution medication prescribing clinicians registered in the database of the National Association of Statutory Health Insurance Physicians – in German “Kassenärztliche Bundesvereinigung (KBV)”. The KBV mailed the invitations on our behalf; 524 responses were received, of which 100 were excluded because they did not provide written informed consent, 42 because clinicians did not currently provide OST, and 63 because they treated three or less patients at the time, leaving 319 clinicians for sampling.

#### Sampling method

Official data on the distribution of OST clinicians in Germany (as of 1.07.2013) was obtained from the Substitution Register. This data was stratified according to German Federal State (GFS; in German “Bundesland”) and the number of patients each clinician was treating (Patients Per Clinician, PPC), the latter consisting of four self-defined categories: (I) 4 – 10 PPC, (II) 11 – 40 PPC, (III) 41 – 80 PPC, (IV) 80 or more PPC. When clinicians replied to the study invitation, they were asked to indicate the number of OST patients they attend to, in order to be allocated to a PPC category. Implausible PPC values were identified and corrected in consultation with the corresponding clinician.

Stratified random sampling was performed to preserve the strata proportions of the population of OST clinicians within the sample. The sampling scheme was structured, based on the distribution of the total sample of OST clinicians in Germany, according to GFS and PPC. Using the SPSS Complex Samples procedure, pre-defined random subsamples for every GFS and PPC category were selected from the eligible sample (*n* = 319).

On 11.07.2014, a representative sample of 88 clinicians was drawn to arrive at approximately 2500 patients as planned (Table 1). If clinicians drop out of the study, participants will be re-recruited to meet the representativeness criteria. If several clinicians meet the criteria, SPSS will be used to draw one at random, and if no more clinicians meet the criteria, they will be drawn from the nearest PPC category rather than a different GFS to maintain national representativeness.

**Table 1:** Distribution of OST clinicians by German Federal State and patients per clinician

	Distribution of OST clinicians in Germany with a qualification for addiction therapy	Distribution of the preliminary sample of OST clinicians in the present study (as of 11.07.2014)	
German Federal State (GFS)	%	n	%
Baden-Württemberg	13.6	11	12.5
Bayern	9.2	8	9.1
Berlin	6.2	5	5.7
Brandenburg	0.3	1	1.1
Bremen	3.0	3	3.4
Hamburg	4.2	4	4.5
Hessen	9.3	8	9.1
Mecklenburg-Vorpommern	0.6	1	1.1
Niedersachsen	9.9	7	8.0
Nordrhein-Westfalen	32.1	27	30.7
Rheinland-Pfalz	3.1	4	4.5
Saarland	0.8	0	0.0
Sachsen	0.8	2	2.3
Sachsen-Anhalt	1.1	1	1.1
Schleswig-Holstein	4.9	5	5.7
Thüringen	0.9	1	1.1
Total	100.0	88	100.0
Patients per clinician (PPC)	%	*n*	%
4–10	16.7	13	14.8
11–40	44.1	38	43.2
41–80	26.9	24	27.3
>80	12.3	13	14.8
Total	100.0	88	100.0

#### Patient recruitment

Patients of participating clinicians are included in the study if they fulfil the eligibility criteria. To avoid clinicians with a large number of OST patients from dominating the study sample, a maximum of 60 patients per clinician is employed. Thus for each clinician with more than 60 patients, ECHO-study research staff draws a random sample of 60 patients.

### Procedure

Once a representative sample of OST clinicians is drawn, written informed consent and coded patient lists are obtained from all clinicians. The codes consist of patients’ initials, date of birth and gender and are used to identify questionnaires when they are sent back to the research centre. Clinicians also indicate which patients started a course of HCV treatment up to 12 months prior to baseline, so that the research staff can send the follow-up questionnaires to the correct patients. If clinicians have more than 60 PPC, research staff draws 60 patients as described above and sends the lists of selected patients back to the clinicians.

Next, study materials are sent and explained to participating clinicians. Data collection begins after patients provide written informed consent. The follow-up phase starts 12 months after study initiation, which may be a different point in time for each patient. In case clinicians do not provide written informed consent, stop communicating to the research staff, or do not collect data, ECHO-study research staff will try to communicate with OST clinicians to understand the reason for their inactivity (e.g., they forgot about the study). If these difficulties are not resolved, the clinician will be excluded and another clinician will be recruited according to the same representativeness criteria.

#### Monitoring framework and quality of data

A 2-level monitoring framework is implemented to improve the quality of data. Monitoring level 1 ensures data completeness. Before data collection begins, ECHO-study research staff briefs clinicians and medical assistants on the study process, explains the material, and trains them in proper documentation. Throughout the study, research staff stays in contact with clinicians and medical staff via telephone calls and personal visits to answer any questions and to ensure participation and complete documentation. Clinicians and medical assistants are responsible for complete and accurate data collection, because data is gathered during routine patient care. Research staff checks the data for plausibility and completeness and may ask clinicians and medical staff to complete or correct missing or implausible data. Research staff merely has access to pseudonymized data at this stage.

In monitoring level 2, extended in-house source data verification (SDV) is conducted to ensure accuracy of recorded data. SDV is performed on 20 % of the patient sample of 25 % of clinicians. The more patients a clinician attends to, the more patients from that clinician are selected for SDV. Trained staff members that are not otherwise involved in the ECHO-study make use of an SDV form and check the data for accuracy. If necessary, they report back to clinicians to clarify and correct implausible or incorrect data entries in the best possible way. All data is stored electronically and safely at the research centre.

### Variables

Clinician and patient questionnaires are administered at baseline. Medical staff can complete parts of the clinician questionnaire, including objective information (e.g., HCV diagnostics) but not subjective ratings (e.g., problem severity in various life areas). Clinicians will complete the follow-up questionnaire for patients that started HCV treatment up to 12 months prior to baseline and for patients that were HCV negative at baseline. Table 2 provides an overview of all instruments.

**Table 2:** An overview of instruments used in the patient and clinician questionnaires

	Sections	Instruments
Patient questionnaire	Sociodemographics	Sociodemographics
	(Health-related) Quality of life	QOL; SF-12
	Physical and mental health	OTI-HSS; BSI-18
	Infectious diseases	Knowledge, needs and attitudes; HCV treatment and treatment experiences
	Autonomy preference	API-Dm
	Satisfaction with care	ZAPA
Clinician questionnaire	Sociodemographics	Sociodemographics
	Physical and mental health	Somatic and psychiatric diseases; CGI
	Routine patient care data	Substitution treatment; HCV diagnostic and treatment; Consumption of substances
	Patient wellbeing	Problem severity in life areas; GAF
Clinician questionnaire (follow-up)	Routine patient care data	Recent HCV treatment; Diagnostics prior to the recent HCV treatment; Current HCV status

*Note*: *QOL* quality of life, *SF-12* 12-Item Short Form Health Survey, *OTI-HSS* Opiate Treatment Index-Health Scale Score, *BSI-18* Brief Symptom Inventory-18, *API-Dm* Autonomy Preference Index - German modified version, *ZAPA* Satisfaction in Outpatient Care – Patient Participation, *CGI* Clinical Global Impression, *GAF* Global Assessment of Functioning

#### Patient questionnaire

##### *A. Sociodemographics*

Sociodemographic factors include sex, age, citizenship, cultural background, relationship, children, housing, and employment.

##### *B. Quality of life (QOL)*

In this self-made questionnaire, patients rate their satisfaction with 10 life domains (employment, relationship, family, leisure and social contacts, finances, housing, physical health, mental wellbeing, substitution treatment, coping with addiction) on a 6-point Likert scale.

##### *C. 12-Item Short Form Health Survey (SF-12)*

The SF-12 is a 12-item health-related quality of life (HRQOL) scale [35] developed to measure functional health and wellbeing from the patient perspective. Physical and mental composite scores are calculated.

##### *D. Opiate Treatment Index Health Symptoms Scale (OTI-HSS) and OTI-HSS-extended*

The Health Symptoms Scale of the OTI [36] is a subjective measure of physical health, comprising a checklist of 50 symptoms for women and 48 for men across 8 areas of health in which opioid users typically experience problems: general (14 items), injection-related (5 items), cardio/respiratory (9 items), genitourinary (4 items), gynaecological (2 items), musculoskeletal (3 items), neurological (10 items), and gastrointestinal (5 items). Patients indicate whether they experienced symptoms during the past 30 days. The total score is the sum of reported symptoms, with higher scores indicating poorer health. A sub-sample of patients receives the OTI-HSS with a self-added 5-point Likert scale concerning symptom burden (1 = *not at all*; 5 = *very strong*), the OTI-HSS-extended.

##### *E. Brief Symptom Inventory-18 (BSI-18)*

The BSI-18 is a self-report measure of psychological distress [37]. The burden of 18 physical and psychological symptoms is rated on a 5-point Likert scale (1 = *not at all*; 5 = *very much*) with regard to the preceding 7 days and yields 3 sub-scores for depression, anxiety, and somatization, and an overall Global Severity Index.

##### *F. Infectious diseases: Knowledge, needs, and attitudes*

This self-made questionnaire is designed to assess patients’ knowledge of their infectious disease status and of HCV treatment related services, as well as patient needs and attitudes toward HCV infections. Specifically, patients indicate their current infection status (Tuberculosis, Human immunodeficiency virus (HIV), Hepatitis A, B, and C Virus) and time elapsed since the last test. They indicate which HCV treatment-related services are offered in their OST practice (4 items) and time elapsed since they last talked to their clinician about HCV. Furthermore, patients indicate whether they have questions about HCV (5 items; e.g., ‘Prevention of HCV’), how strongly they agree with statements (4 items; e.g., ‘It is important to me to be free of HCV’) and whether statements apply to them (3 items; e.g., ‘My clinician recommended HCV treatment to me’).

##### *G. HCV Treatment and Treatment Experience*

Patients indicate whether they are currently in or plan to start HCV treatment, and whether they would take up HCV treatment. Provided they are or have been in HCV treatment, patients are queried about treatment success, treatment provider (e.g., their OST clinician), side effects, and satisfaction with information given in preparation to treatment.

##### *H. Autonomy Preference Index - German modified version (API-Dm)*

The API-Dm is a measure of patient preferences for decision-making and information seeking [38]. Sub-scales *participation preference* (4 items) and *information preference* (7 items) are rated on a 5-point Likert scale (1 = *very much in favour*; 5 = *very much against*) and scored separately. The two total scores are transformed to a range from 0 to 100, with higher scores corresponding to a higher preference for decision-making and information seeking.

##### *I. Satisfaction in Outpatient Care – Patient Participation (ZAPA)*

The ZAPA is a German scale measuring satisfaction in outpatient care with a focus on patient participation (‘Zufriedenheit in der ambulanten Versorgung – Schwerpunkt Patientenbeteiligung’, [39]. Three items (trust in the clinician, satisfaction with information given, and quality of the treatment) are rated on a 4-point Likert scale. The fourth item ‘satisfaction with participation’ is omitted in the present study, because it is covered by API-Dm. The ZAPA is administered twice to assess satisfaction with OST and satisfaction with HCV treatment.

#### Clinician questionnaire

##### *A. Sociodemographics*

Sociodemographic factors include patient’s sex, date of birth, height, weight, citizenship, age at onset of opioid dependence.

##### *B. Substitution treatment*

OST items comprise date of OST initiation in the present clinic, whether the patient is in OST for the first time, and current substitution medication and dose.

##### *C. HCV diagnostic and treatment*

Patient’s most recent anti-HCV test and HCV-RNA test results (including viral load, HCV genotype(−subtype), and duration of chronic HCV infection) and dates of test results are recorded from the participant’s chart. Clinicians are reminded of the HCV treatment guidelines in Germany, which recommend yearly HCV testing in this high-risk population. Further, clinicians report patient’s past receipt of HCV treatment, treatment start and end date, treatment outcome and, if applicable, reasons for treatment termination.

##### *D. Somatic and psychiatric diseases*

Patients’ health over the past 6 months is assessed by means of a checklist of somatic diseases (Hepatitis B virus (HBV), HIV, Tuberculosis, Chronic obstructive pulmonary (COPD), or Other) and psychiatric disorders or symptoms (Depression, Anxiety, Psychotic disorder/symptoms, Posttraumatic stress disorder (PTSD), Psychopharmacological treatment, or Other).

##### *E. Consumption of substances*

Recent consumption of cocaine, benzodiazepines, heroin, buprenorphine, amphetamine and cannabis is examined using the last three urine sample results.

##### *F. Problem severity in life areas*

Clinicians provide a subjective evaluation of patient’s current problem severity in various life areas. Twelve life domains (consumption of illegal drugs, consumption of non-prescribed medication, alcohol consumption, physical health, mental wellbeing, housing, partnership or family, friends, leisure, employment or education, finances, legal situation) are rated on a 3-point Likert scale (1 = *no problem*, 2 = *small problem*, 3 = *large problem*).

##### *G. Clinical Global Impression (CGI) scale*

The present study uses a shortened, adjusted version of the CGI scale [40]. Clinicians estimate the current severity of patients’ mental illness and the degree of improvement since the start of OST on a 7-point Likert scale; the two items do not yield a global score.

##### *H. Global Assessment of Functioning (GAF) scale*

The GAF Scale [41] is an observer-rated single-item rating of functioning. Patients’ psychological, social and occupational functioning during the past 12 months is assessed on a mental health-illness continuum from 100 to 0 (100 – 91 = *No Symptoms*; 10 – 1 = *Persistent Danger or Non-functionality*).

#### Clinician questionnaire (follow-up)

##### *A. Recent HCV treatment*

We examine the course of HCV treatment, which started up to 12 months prior to study baseline: Start and end date of the course of HCV treatment, treatment outcome, whether the patient completed treatment regularly or terminated treatment early, and, if applicable, reasons for terminating treatment. Furthermore, test results (viral load) of the last HCV-RNA test prior to HCV treatment and of the most recent HCV-RNA test post-treatment; whether the patient experienced clinically relevant complications due to leukopenia, thrombocytopenia, anaemia, or other HCV treatment side effects; type of HCV medication; and accompanying medication (antiretroviral therapy (HIV), benzodiazepine, antidepressants, other psychotropics).

##### *B. Diagnostics prior to the recent HCV treatment*

HCV genotype and subtype, duration of the chronic HCV infection, degree of fibrosis, and whether the patient had received HCV treatment before.

##### *C. Current HCV status*

The current HCV status is recorded from the participant’s chart. Clinicians are reminded of the HCV treatment guidelines in Germany, which recommend yearly HCV testing in this high-risk population.

### Planned statistical analysis

HCV prevalence will be calculated based on data from all OST patients at baseline. Additionally, RNA confirmation tests will provide an estimate of the distribution of HCV genotypes and subtypes. To calculate the incidence rate per person-years, the expected subsample of approximately 800 HCV seronegative patients (given a prevalence rate of 68 % anti-HCV seropositive patients, see ‘Sample Size’) will be observed over the course of 12 months. Factors influencing HCV treatment uptake (e.g., physical and mental health) and seroconversion (e.g., concomitant drug use) will be tested by means of bivariate and multivariate analyses (e.g., logistic regression) based on the scale levels of the respective variables. If patients terminate or drop out of OST or no longer wish to participate in the study, the reasons and circumstances will be documented. Dropouts will be analysed in an attempt to identify possible predictors for an early end of treatment.

### Ethical considerations

Patients in OST are often seen as ‘difficult patients’ due to comorbidities and cognitive impairments, while OST clinicians are busy in everyday clinical practice. Therefore data collection is integrated into routine patient care to minimize the burden of the study to both patients and clinicians. Clinicians selected for the study receive extensive support from research centre staff throughout the study process, and both patients and clinicians receive adequate remuneration.

Written informed consent is obtained from patients and clinicians. Patient data is documented in the study centres (OST practices); ECHO-study research staff only has access to pseudonymized data and no patient-related data. A trained staff member who is not otherwise involved in the ECHO study conducts the SDV. Data is exclusively handled in the research centre and stored safely in a protected database, which adheres to the legal data protection regulations. Ethical approval was granted by the Ethics Committee of the Medical Association of Hamburg, Ref. PV4603, and by each local Ethics Committee in Germany.

## Discussion

Injecting drug use remains the primary mode of HCV acquisition in the Western world [42]. Diagnosis, treatment and monitoring of HCV infection among drug users must be increased and improved in response to this endemic. OST may be an important strategy in reducing the rate of HCV infection among drug users, but evidence for its effectiveness remains weak to date [18–22]. ECHO will be the first study to provide representative data on the prevalence and incidence of HCV infection among OST patients, as well as factors associated with HCV treatment uptake and seroconversion. As such, it will extend the literature by demonstrating the real-life impact of OST on HCV infection at a national level.

The present study will provide important information for developing strategies to address HCV-related disease burden in this population. Specifically, health care research on HCV prevention and harm reduction strategies will benefit from this study’s findings. Furthermore, the information gained from this project will be essential for HCV modelling exercises, which can inform health policy. The data may be used to model projections of HCV prevalence over the next decades based upon current levels of HCV treatment.

A better understanding of barriers and facilitators to HCV treatment uptake and seroconversion among people receiving OST is important, particularly in light of the new interferon-free HCV treatment regimens. New DAA-based regimens have the potential of substantially reducing the prevalence of HCV infection, however they are very costly and health care providers are reluctant to prescribe them to drug users, who are considered a high-risk group for reinfection. Knowing what factors maximize the success of HCV therapy in OST patients is essential for convincing service providers to offer the new, effective HCV therapy to people receiving OST.

Beyond our main research questions, we will examine a range of covariates and associated topics, such as QOL and shared decision-making. QOL is increasingly acknowledged as an important treatment outcome criterion in OST. Yet most studies merely measure QOL at the beginning of OST. The ECHO study will be the first to deliver cross-sectional, representative data on the QOL for an entire population of patients in OST. Furthermore, patient-centred care is recognized as a measure to improve health care by empowering patients to be actively involved in their treatment. Yet this approach is not applied in OST. ECHO will be one of the first studies to explore OST patients’ preferences in shared decision-making, satisfaction in patient care, and needs and attitudes concerning treatment.

The present study appears feasible, as we have been able to draw a clinician sample according to the representativeness criteria GFS and PPC up until manuscript submission, and our monitoring framework appears effective. While questionnaires are occasionally incomplete, the implemented monitoring system is efficient and ensures complete data sets. We hope this is encouraging to researchers in other countries to pursue similar investigations.

The study has some anticipated limitations. The sample may be subject to selection bias, as only 319 of 2691 OST clinicians were willing and eligible to take part in the ECHO study and clinicians have been dropping out of the study since. Particularly clinicians from small practices seem unwilling to participate in the present study. A similar pattern was observed in a study in which clinicians from small OST centres did not regularly test for HCV infection in patients [14]. The representability of the sample may be compromised if too many clinicians drop out.

Further, the sample size calculation assumes a 1-year follow-up of all individuals and does not consider potential dropouts. Incidence estimates may be biased if only a small sub-sample returns for follow up. However, Germany has one of the highest retention rates of OST in the world [43]. The present study will provide first insights into HCV incidence among OST patients. All potential limitations will be discussed in following publications.

## Abbreviations

API-Dm: Autonomy Preference Index - German modified version
AWMF: Association of the Scientific Medical Societies in Germany
BfArM: Federal Institute for Drugs and Medical Devices
BSI-18: Brief Symptom Inventory-18
BtMVV: Ordinance on the Prescription, Dispensing and Proof of the Disposition of Narcotic Drugs
CGI: Clinical Global Impression
COPD: Chronic obstructive pulmonary
DAA: Direct-acting antivirals
ECHO: Epidemiology of Hepatitis C Virus Infection among People Receiving Opioid Substitution Therapy
GAF: Global Assessment of Functioning
GFS: German Federal State
HBV: Hepatitis B virus
HCV: Hepatitis C virus
HIV: Human immunodeficiency virus
HRQOL: Health-related quality of life
ICD-10: International Statistical Classification of Diseases and Related Health Problems, 10^th^ revision
KBV: National Association of Statutory Health Insurance Physicians
OST: Opioid substitution therapy
OTI-HSS: Opiate Treatment Index Health Symptoms Scale
PPC: Patients Per Clinician
PTSD: Posttraumatic stress disorder
PWID: People who inject drugs
QOL: Quality of life
SDV: Source data verification
SF-12: 12-Item Short Form Health Survey
ZAPA: Satisfaction in Outpatient Care – Patient Participation
